# Cardiometabolic risk factors among patients with tuberculosis attending tuberculosis treatment centers in Nepal

**DOI:** 10.1186/s12889-020-09472-0

**Published:** 2020-09-05

**Authors:** Indra Prasad Poudyal, Pratik Khanal, Shiva Raj Mishra, Milan Malla, Prakash Poudel, Raj Kumar Jha, Anil Phuyal, Abiral Barakoti, Bipin Adhikari

**Affiliations:** 1 Institute of Medicine, Tribhuvan University, Maharajgunj, Kathmandu, Nepal; 2 Nepal Development Society, Bharatpur, Chitwan Nepal; 3 Queensland University, Brisbane, Queensland Australia; 4 Department of Health Services, Ministry of Health and Population, Kathmandu, Nepal; 5 Centre for Tropical Medicine and Global Health, Nuffield Department of Medicine, University of Oxford, Oxford, UK

**Keywords:** Tuberculosis, Cardiometabolic risk factors, Non-communicable diseases

## Abstract

**Background:**

The co-morbidity of cardiometabolic diseases in patients with Tuberculosis adds a significant burden in current health systems in developing countries including Nepal. The main objective of this study was to explore cardiometabolic risk factors among patients with Tuberculosis.

**Methods:**

This was a cross-sectional study conducted among patients with tuberculosis in 12 tuberculosis treatment centers from eight districts of Nepal between May and July 2017. Interviews with participants were conducted using a structured questionnaire and were supplemented by anthropometric measurements and on-site blood glucose tests. Data were analyzed using descriptive and inferential statistics.

**Results:**

Among 221 study participants, 138 (62.4%) had new smear-positive pulmonary tuberculosis, 24 (10.9%) had new smear-negative pulmonary tuberculosis and 34 (15.4%) had new extra- pulmonary tuberculosis. Overall, 43.1% of the patients with tuberculosis had at least one cardiometabolic risk factor. The prevalence of at least one cardiometabolic risk factor was more in male than female (47.8% versus 33.8%). Prevalence of tobacco (18.9% versus 4.8%), and alcohol (12.6% versus 6.5%) use was proportionately higher in male compared to female. The prevalence of hypertension (17% vs. 21%) and obesity (11.9% vs. 12.9%) was lower in male compared to females. Female (AOR = 0.47; CI: 0.23–0.94), those from Gandaki Province (AOR = 0.32; CI: 0.13–0.79) and literate (AOR = 0.49; CI: 0.25–0.96) had reduced risk of cardiometabolic disease risk factors.

**Conclusions:**

This study highlights the role of gender and socio-demographic characteristics associated with the risk of cardiometabolic diseases in patients with Tuberculosis. The findings from this study can guide medical practitioners and policy makers to consider clinical suspicion, diagnosis and treatment. National treatment guideline can benefit by integrating the management of non-communicable diseases in Tuberculosis treatment centers.

## Background

Tuberculosis (TB) constitutes a top ten causes of mortality globally and resulted in 1.49 million deaths in 2018 [1]. Low and middle-income countries (LMICs) with poor universal health coverage share a disproportionate burden of morbidity and mortality due to TB despite the availability of effective anti-tubercular therapy [1, 2]. The highest incidence (2/3rd of the global burden) of new TB cases was reported from South East Asia and the Western Pacific region followed by the African region, which shared a fourth of the global incidence. It is estimated that 95% of all TB cases and 98% of all TB deaths occur in South East Asia and Africa [2]. Various factors synergize the burden of morbidity and mortality related to TB in developing countries that includes poverty, poor public health system, and co-morbidities [3]. Thus, TB continues to be a persisting challenge in global health [4].

Among an estimated 10 million new TB cases worldwide in 2018, 5.7 million (57%) were found in men, 3.2 million (32%) in women and 1.1 million (11%) in children [5]. In Nepal, almost half of the total population (45%) is infected with TB and the most affected population (60%) is productive age group (18–45 years). Annually, 44,000 people develop active TB, and half of which is Pulmonary TB with around 7000 deaths every year [6].

Together with the burden of TB [7], developing countries suffer from an added burden due to the rising epidemic of non-communicable diseases (NCDs) mainly cardiovascular diseases (CVDs). The CVDs are caused by a combination of cardiometabolic risk factors such as tobacco use, physical inactivity, cholesterol, diabetes, obesity and hypertension [8, 9]. In Nepal, the epidemiological shift has transformed the burden of diseases from communicable to NCDs; the latter contribute to around two-thirds of mortality [10]. The CVDs and its risk factors are prominent in Nepal [11–14]. According to Nepal’s Global Burden of Disease Report 2017, Ischemic Heart Disease is the topmost cause of death while cerebrovascular hemorrhage is the fifth major cause of deaths [10]. Studies have shown that population from rural communities in Nepal have inadequate knowledge, attitude and behavior related to cardiovascular health; and are also at equal risk of CVDs as their urban counterparts [12–15]. Besides, the health system readiness for the management of CVDs and other NCDs is sub-optimal in Nepal [10, 16–18].

While there are few studies conducted on cardiometabolic risk factors among migrants [19], adult population [20, 21] and people living with HIV [22] in Nepal and other developing countries, we could not find any studies on TB patients. The consequence of diabetes, hypertension as well as other cardiometabolic risk factors among patients with TB is under-appreciated. The dearth of evidence has further affected the coordination between TB and NCD control program in Nepal. This study aimed to explore the prevalence of cardiometabolic risk factors among patients with TB attending DOTS centers of Nepal.

## Methods

### Study design and study setting

This was a facility-based cross-sectional study conducted in 12 TB centers (DOTS centers) in eight out of 77 districts of Nepal between May and July 2017. The study districts were Morang in Province 1, Dhading in Province 3, Baglung and Tanahaun in Gandaki province, Rolpa in Province 5 and Kailali, Dadeldhura and Doti in Sudurpaschim province. These districts and DOTS centers were purposively selected to ensure inclusion of the urban and non-urban settings of the country. All the patients visiting the treatment centers during the data collection period were recruited for the study purpose.

A total of 238 patients with TB visiting DOTS centers for anti-tubercular therapy during the study period and meeting the eligibility criteria participated in this study. The participants were selected from DOTS clinics of Primary Health Centers (PHCs) and government hospitals of the country. The inclusion criteria for the participants included: (1) aged 15 years and above; (2) newly diagnosed TB or currently under anti-tubercular medication; and (3) those willing to give written informed consent to participate in the study. Pregnant women and lactating mothers were excluded from the study considering associated gestational diabetes mellitus.

### Data collection measures

Patients were interviewed at the DOTS clinic through a structured questionnaire by paramedics or medical officers who were trained to fill the questionnaires. Information about socio-demographic and behavioral characteristics was obtained through interviewing study participants while information on TB category was collected from the patient card. Data on anthropometric measurements and blood pressure assessment were collected using the standard method as described in the WHO STEPS survey [23]. Random blood sugar level was measured in the laboratory by Glucose oxidase– peroxidase method for undiagnosed cases and the value of 200 mg/dl or more for diabetes and 140 mg/dl or more for pre-diabetes as recommended by the American Diabetes Association (ADA) [24]. Current tobacco use was described as those who have used smoked or consumed smokeless tobacco within 30 days while current alcohol use was described as those who have at least drank once in the past 30 days. Although cholesterol level is an important cardiometabolic risk factor, lipid profile test was not performed in this study because such tests were not available at the primary health care settings.

The questionnaire for data collection was developed based on an extensive literature review. Questionnaire related to socio-demographic characteristics was adapted from Nepal Demographic Health Survey 2016 [25] while information related to behavioral, clinical and anthropometric characteristics was based on the WHO STEPS survey [23].

### Study variables

Socio-demographic variables included data on age, sex, ethnicity, geographical location, educational status and occupation. Behavioral variables included current alcohol use and current tobacco use. Clinical variables included diabetes status, hypertension status and prevalence of HIV/AIDS. Anthropometric variables included measurement of Body Mass Index (BMI). The hypertension status has been presented using both American Heart Association (AHA) and the Joint National Committee for hypertension (JNC) criteria.

For inferential analysis, the prevalence of at least one cardiometabolic risk factor was the dependent variable and all other variables were considered as independent variables. In this study, the presence of any risk factors such as alcohol use, tobacco use, diabetes, and hypertension based on the JNC criteria; and overweight/obesity was considered as the presence of cardiometabolic risk factors.

### Data management

Data were entered in EpiData Version 3.1 and was analyzed using IBM SPSS version 20. Continuous variables were summarized as mean with standard deviation (SD). Categorical variables were expressed as frequencies and percentages and Chi-squared test was performed to compare the proportions. A logistic regression analysis was used to explore the association between socio-demographic variables (independent variables) with the presence of at least one cardiometabolic risk factor (dependent variable). Both unadjusted and adjusted odds ratio (AOR) were analyzed. A significant statistical association were considered when *p* values were < 0.05 with 95% Confidence Interval (CI).

## Results

A total of 238 patients with TB participated in the study. Among them, 221 participants with complete information were retained in the analysis.

### Treatment category of patients with TB

Among the study participants, 62.4% (138/221) had new smear-positive pulmonary TB, 10.9% (24/221) had new smear-negative pulmonary TB and 15.4% (34/221) had new extra-pulmonary TB. The proportion of relapse cases, defaulters and those under treatment failure category among the total cases was 9.0% (20/221), 1.8% (4/221) and 0.5% (1/221) respectively (Table 1).

**Table 1:** Tuberculosis category of the study participants (*n* = 221)

TB status	Frequency	Percentage
New smear Positive Pulmonary TB	138	62.4
New smear Negative Pulmonary TB	24	10.9
New Extra Pulmonary TB	34	15.4
Relapse	20	9.0
Treatment failure	1	0.5
Defaulter	4	1.8

### Socio-demographic characteristics

The mean age of the participants (±SD) was 45.19 ± 17.33 years. In this study, 71.9% of the study participants were male, 30.8% were illiterate and 48.9% were engaged in agriculture as their primary occupation. Major proportion of participants belonged to the *Janajati* ethnic group (41.6%) followed by *Brahmin/Chhetri* (32.1%) (Table 2).

**Table 2:** Socio-demographic and behavioral characteristics and comorbidities among patients with TB (*n* = 221)

Variables	Frequency	Percentage (%)
Socio-demographic variables		
Age		
Less than 20	12	5.4
20–39	74	33.5
40–59	81	36.7
60 and above	54	24.4
Sex		
Male	159	71.9
Female	62	28.1
Ethnicity		
Janajati	92	41.6
Brahmin/Chhetri	71	32.1
Dalit	40	18.1
Others (Madheshi, Muslim, Thakuri, Sanyasi)	18	8.2
Education level		
Illiterate	68	30.8
Informal	39	17.6
Primary	32	14.5
Secondary	55	24.9
Higher secondary and above	27	12.2
Occupation		
Agriculture	108	48.9
Unskilled manual	54	24.4
Skilled manual (Professional, technical, clerical)	40	18.1
Sales and services	19	8.6
Province		
One	45	20.4
Three and Five	35	15.8
Gandaki	54	24.4
Sudurpaschim	87	39.4

### Behavioral and clinical characteristics

The proportion of current tobacco and alcohol use among the study participants was 14.9% (33/221) and 10.9% (24/221) respectively. The mean BMI of the participants (±SD) was 19.88 ± 4.13 kg/ m^2^ with 43.0% underweight, 9.9% overweight and 2.3% obese (Table 3). The patient report showed that 3.6% (8/221) of the patients with TB had HIV. The proportion of pre-diabetics was 15.8% (35/221) and diabetics were 6.3% (14/221) (Table 3). The mean blood glucose of the participants (±SD) was 114 ± 46.13 mg/dl.

**Table 3:** Behavioral and clinical characteristics among patients with TB (*n* = 221)

Variables	Frequency	Percentage (%)
Anthropometric and Behavioral characteristics		
BMI (kg/m^2^)		
Too thin for height (< 18.5)	95	43.0
Normal (18.5–24.9)	99	44.8
Overweight (24.9–29.9)	22	9.9
Obese (> 30)	5	2.3
Tobacco use		
Current	33	14.9
Past	119	53.8
Never	69	31.2
Alcohol use		
Current	24	10.9
Past	123	55.7
Never	74	33.5
Clinical characteristics		
HIV		
Positive	8	3.6
Negative	213	96.4
Diabetic mellitus^a^ (≥140 mg/dl)		
Yes	35	15.8
No	186	84.2
Diabetes mellitus (≥200 mg/dl)		
Yes	14	6.3
No	207	93.7
Hypertension (JNC^b^)		
Normal	123	55.7
Pre-hypertension	58	26.2
Stage I Hypertension	26	11.8
Stage II Hypertension	3	1.4
Hypertensive under medication	11	5.0
Hypertension (AHA^c^)		
Normal	123	55.7
Elevated BP	16	7.2
Stage I Hypertension	42	19.0
Stage II Hypertension	29	13.1
Hypertensive under medication	11	5.0
Family history of Hypertension		
Yes	11	5.0
No	96	43.4
Unknown	114	51.6

^a^including prediabetes

^b^JNC classification

^c^AHA classification

The mean systolic and diastolic blood pressure (±SD) was 112.5 ± 18.1 mmHg and 72.2 ± 11.8 mmHg respectively. The prevalence of hypertension according to the AHA classification was 37.1% (82/221) and according to the JNC classification was 18.2% (40/221). Among the study participants, 5% (11/221) were under the anti-hypertensive medication and 5% (11/221) had positive first-degree family history for hypertension (Table 3).

Among those who were under anti-hypertensive medications, 72.7% (8/11) had uncontrolled blood pressure according to AHA classification while 63.6% (7/11) had uncontrolled blood pressure according to JNC classification (Data not shown).

### Cardiometabolic risk factors among patients with TB

Overall, 43.1% (97/221) of the patients with TB had at least one cardiometabolic risk factor. The prevalence of at least one cardiometabolic risk factor was more in male than female (47.8% versus 33.8%). The proportion of patients with TB currently using tobacco, alcohol and having diabetes was higher in males than females while the proportion was higher in females as compared to males for hypertension and being overweight or obese (Table 4). While examining the factors associated with the prevalence of at least one cardiometabolic risk factor, those from Gandaki province (AOR = 0.32; CI: 0.13–0.79) had lower odds as compared to province one. Female (AOR = 0.47; CI: 0.23–0.94) and literate (AOR = 0.49; CI: 0.25–0.96) study participants had a lower chance of having cardiometabolic risk factor as compared to male and illiterate study participants (Table 5).

**Table 4:** Clustering of cardiometabolic risk factors and their socio-demographic correlates among patients with TB (*n* = 221)

	Overweight/obesity	Tobacco	Alcohol	Diabetes	Hypertension	≥1 risk factors
Variables	n(%)	n(%)	n(%)	n(%)	n(%)	n(%)
Total^a^	27 (12.2)	33 (14.9)	24 (10.9)	14 (6.3)	40 (18.1)	97 (43.1)
**Male**						
**Age**						
Less than 20	0 (0)	0 (0)	0 (0)	0 (0)	0 (0)	0 (0)
20–39	6 (31.6)	7 (23.3)	7 (35.0)	1 (8.3)	7 (25.9)	22 (28.9)
40–59	12 (63.1)	14 (46.7)	5 (25.0)	3 (25.0)	14 (51.9)	32 (42.2)
60 and above	1 (5.3)	9 (30.0)	8 (40.0)	8 (66.7)	6 (22.2)	22 (28.9)
**Ethnicity**						
Janajati	8 (42.1)	8 (26.7)	6 (30.0)	6 (50.0)	8 (29.6)	27 (35.6)
Brahmin/Chhetri	6 (31.6)	12 (40.0)	6 (30.0)	3 (25.0)	8 (29.6)	27 (35.6)
Dalit	3 (15.8)	6 (20.0)	6 (30.0)	2 (16.7)	9 (33.3)	15 (19.7)
Others	2 (10.5)	3 (13.3)	2 (10.0)	1 (8.3)	2 (7.5)	7 (9.1)
**Education level**						
Illiterate	2 (10.5)	15 (50.0)	13 (65.0)	6 (50.0)	11 (40.7)	28 (36.8)
Informal	6 (31.6)	6 (20.0)	1 (5.0)	2 (16.7)	7 (25.9)	15 (19.7)
Primary	3 (15.7)	4 (13.3)	1 (5.0)	1 (8.3)	1 (3.8)	10 (13.2)
Secondary	4 (21.1)	4 (13.3)	5 (25.0)	2 (16.7)	5 (18.5)	14 (18.4)
Higher secondary and above	4 (21.1)	1 (3.4)	0 (0)	1 (8.3)	3 (11.1)	9 (11.9)
**Occupation**						
Agriculture	7 (36.8)	17 (56.7)	13 (65.0)	10 (83.4)	12 (44.4)	41 (53.9)
Unskilled manual	5 (26.3)	5 (16.7)	4 (20.0)	1 (8.3)	6 (22.2)	15 (19.8)
Skilled manual	4 (21.1)	4 (13.3)	1 (5.0)	1 (8.3)	5 (18.6)	11 (14.5)
Sales and services	3 (15.8)	4 (13.3)	2 (10.0)	0 (0)	4 (14.8)	9 (11.8)
**Province**						
One	5 (26.3)	7 (23.3)	4 (20.0)	1 (8.3)	9 (33.3)	16 (21.1)
Three and Five	1 (5.3)	7 (23.3)	7 (35.0)	2 (16.7)	3 (11.1)	13 (17.1)
Gandaki	8 (42.1)	4 (13.3)	1 (5.0)	1 (8.3)	6 (22.3)	14 (18.4)
Sudurpaschim	5 (26.3)	12 (40.1)	8 (40.0)	8 (66.7)	9 (33.3)	33 (43.4)
**Male total**^**a**^	**19 (11.9)**	**30 (18.9)**	**20 (12.6)**	**12 (7.5)**	**27 (17.0)**	**76 (47.8)**
**Female**						
**Age**						
Less than 20	0 (0)	0 (0)	0 (0)	0 (0)	0 (0)	0 (0)
20–39	5 (62.5)	0 (0)	1 (25.0)	0 (0)	4 (30.8)	7 (33.3)
40–59	2 (25.0)	3 (100.0)	2 (50.0)	1 (50.0)	6 (46.1)	10 (47.6)
60 and above	1 (12.5)	0 (0)	1 (25.0)	1 (50.0)	3 (23.1)	4 (19.1)
**Ethnicity**						
Janajati	5 (62.5)	1 (33.3)	2 (50.0)	1 (50.0)	7 (53.8)	10 (47.6)
Brahmin/Chhetri	2 (25.0)	0 (0)	0 (0)	0 (0)	3 (23.1)	5 (23.8)
Dalit	1 (12.5)	2 (66.7)	2 (50.0)	1 (50.0)	3 (23.1)	6 (28.6)
Others	0 (0)	0 (0)	0 (0)	0 (0)	0 (0)	0 (0)
**Education level**						
Illiterate	2 (25.0)	3 (100)	4 (100.0)	2 (100.0)	6 (46.2)	12 (57.2)
Informal	2 (25.0)	0 (0)	0 (0)	0 (0)	4 (30.8)	4 (19.0)
Primary	0 (0)	0 (0)	0 (0)	0 (0)	0 (0)	0 (0)
Secondary	3 (37.5)	0 (0)	0 (0)	0 (0)	3 (23.0)	4 (19.0)
Higher secondary and above	1 (12.5)	0 (0)	0 (0)	0 (0)	0 (0)	1 (4.8)
**Occupation**						
Agriculture	4 (50.0)	2 (66.7)	3 (75.0)	1 (50.0)	11 (84.6)	13 (61.8)
Unskilled manual	3 (37.5)	1 (33.3)	1 (25.0)	1 (50.0)	1 (7.7)	6 (28.6)
Skilled manual	1 (12.5)	0 (0)	0 (0)	0 (0)	0 (0)	1 (4.8)
Sales and services	0 (0)	0 (0)	0 (0)	0 (0)	1 (7.7)	1 (4.8)
**Provinces**						
One	4 (50.0)	2 (66.7)	3 (75.0)	0 (0)	5 (38.5)	9 (42.9)
Three and Five	2 (25.0)	0 (0)	0 (0)	1 (50.0)	1 (7.7)	3 (14.3)
Gandaki	1 (12.5)	0 (0)	0 (0)	0 (0)	2 (5.3)	2 (9.5)
Sudurpaschim	1 (12.5)	1 (33.3)	1 (25.0)	1 (50.0)	5 (38.5)	7 (33.3)
**Female total**^**a**^	**8 (12.9)**	**3 (4.8)**	**4 (6.5)**	**2 (3.2)**	**13 (21.0)**	**21 (33.9)**

^a^total n(%) of having individual with ≥1 risk factors with and without disaggregation by sex

**Table 5:** Factors associated with at least one cardiometabolic risk factor (*n* = 221)

Variables	≥1 risk factors n(%)	Unadjusted OR (95% CI)	Adjusted OR (95% CI)
**Age**			
Less than 40	29 (29.9)	Ref	Ref
40 and above	68 (70.1)	2.00 (1.14–3.49)*	1.49 (0.77–2.90)
**Sex**			
Male	76 (78.4)	Ref	Ref
Female	21 (21.6)	0.56 (0.34–1.03)	0.47 (0.23–0.94)*
**Ethnicity**			
Dalit	21 (21.6)	Ref	Ref
Janajati	37 (38.1)	0.61 (0.29–1.29)	0.56 (0.25–1.27)
Brahmin/Chhetri	32 (33.0)	0.74 (0.34–1.62)	0.72 (0.31–1.68)
Others	7 (7.3)	0.58 (0.19–1.79)	0.37 (0.11–1.26)
**Education level**			
Illiterate	40 (41.2)	Ref	Ref
Literate	57 (58.8)	0.42 (0.23–0.75)*	0.49 (0.25–0.96)*
**Occupation**			
Agriculture	54 (55.7)	Ref	Ref
Skilled manual	12 (12.4)	0.43 (0.20–0.93)*	0.71 (0.29–1.71)
Sales and Service	10 (10.3)	1.11 (0.42–2.95)	1.30 (0.46–3.70)
Unskilled manual	21 (21.6)	0.64 (0.33–1.24)	0.79 (0.38–1.64)
**Province**			
One	25 (25.8)	Ref	Ref
Three and Five	16 (16.5)	0.67 (0.28–1.64)	0.52 (0.20–1.37)
Gandaki	16 (16.5)	0.34 (0.15–0.77)*	0.32 (0.13–0.79)*
Sudurpaschim	40 (41.2)	0.68 (0.33–1.40)	0.62 (0.28–1.36)

*Significant at *p* < 0.05

## Discussion

### Overall findings

This study identified significant factors affecting the risk of cardiometabolic diseases in patients with TB. Our study findings showed that 43.1% of patients with TB had at least one cardiometabolic risk factor and males were more at risk than females including the behaviors related to consumption of tobacco, and alcohol. Nevertheless, the proportion of hypertension and obesity status was higher in females compared to males. Sex, geographic location and patients’ education level were significantly associated with the risk of cardiometabolic diseases.

### Cardiometabolic risk in males

The preponderance of males in bearing cardiometabolic risk factors highlights their higher proportion of developing TB and thus cardiometabolic diseases. The predominance of males in developing TB echoes with the global and national reports [5, 6] which may partially explain the risk of cardiometabolic diseases among these patients. Nonetheless, the socio-cultural role of males in Nepalese society where their increased exposure to work and occupation, food and life-style related behavior compared to females who may not have similar exposure, further explains the higher risk of cardiometabolic risk factors [19]. One associated factor within this study that sheds light on male’s increased risk of cardiometabolic diseases is the higher consumption of tobacco and alcohol more than females [13, 19]. Socio-culturally in Nepal, males are at the forefront of earning money and managing the household expenditure. Availability of cash, together with the cultural benefits due to patriarchy in Nepalese society can further explain the increased leeway for males that conduces the affordability for the consumption of alcohol and tobacco [13]. These high-risk behaviors are further predicated on other factors such as level of education, occupation and the individual motivation towards a healthy lifestyle. The significantly higher odds of having at least one risk to cardiometabolic diseases in male patients with TB further support our argument.

### Females, obesity and hypertension

The proportion of female patients with TB in this study who had a higher risk of developing hypertension and diabetes resonates with the nationally representative survey where females were found to be the vulnerable population in developing cardiometabolic diseases, [26] and can be explained by the socio-cultural entanglements of female’s role in Nepalese society. Similar to other South Asian nations, females are often housebound, particularly those who are unemployed or are in poor-socio-economic status, and are found to have higher rates of obesity and cardiometabolic risks than male counterparts [27–29]. Females are culturally repressed in their outdoor activities often because of threats of sexual harassments and violence. Such cultural restrictions are further aggravated by lack of urban green spaces, parks and exercise places conducive for physical exercise in South Asia [30]. Embedded in the patriarchal culture of Nepal [31], females are not only burdened by household chores such as cooking, but they are also vulnerable to delayed and irregular eating. Delayed and irregular eating generally stems from cultural and traditional niceties of serving the male members of the family first. Besides, wasting of cooked food in traditional Nepali family (usually devoid of refrigerator) is considered ‘ominous’ which can add pressure to the female members to finish the remaining portion of food. The latter can aggravate irregular and overeating. Women in Nepal are also vulnerable to fasting based on religious and cultural practices. The ramifications of delayed, irregular (over and under) eating and fasting are established to increase the risk of developing obesity, diabetes and hypertension [32]. These socio-culturally shaped behaviors contribute to higher prevalence of cardiometabolic risks in females in Nepal. Though female had significantly lower odds of having at least one cardiometabolic risk factor in this study as compared to males, the greater risk of hypertension and overweight/obesity cannot be neglected.

### Socio-demographic factors and cardiometabolic risk

Other socio-demographic factors that affect higher risk to cardiometabolic diseases are equally important. Although the population from Gandaki province had a lower risk of developing cardiometabolic risk, causal explanations are hard to draw from the geographic location alone. Nevertheless, this may have been due to the socio-demographic characteristics of the population in Gandaki province, such as higher education level, relatively higher affluence, organized urban planning with adequate space for exercise, increased awareness in regards to food and behavior and other socio-ecological factors.

In this study, literate patients with TB showed reduced risk of cardiometabolic diseases and echoes with previous studies from Nepal [29]. Our findings are also consistent with South African study where the risk of cardiometabolic disorders was higher among men, and was lower in those with higher education and socio-economic status [33]. The fact that higher education, in general, might have led to increased awareness regarding the NCDs such as diabetes and hypertension and thus the personal modification in food and lifestyle related behavior could be one of the mechanisms to explain the finding.

### Limitations and further area of research

This cross-sectional study relied on questionnaire-based survey at 12 DOTS centers across Nepal and the results are largely representative for eight districts in Nepal. Nevertheless, the study being cross-sectional and dependent on quantitative assessment, causal explanations of the association for cardiometabolic risks among TB patients could not be adequately explained. In future, qualitative studies using in-depth interviews and focus group discussions with the patients with TB can yield a rich set of data to explain the associated factors with cardiometabolic diseases in this study. Also, further studies can build to explore how the current DOTS centers can increasingly collaborate in the management of co-morbid cardiometabolic conditions with evidence suggesting that risk factors of death among patients with TB are non-infectious co-morbidities as well as alcohol and substance abuse [34]. As TB and HIV prevalence continues to decline in Nepal, operational and health system research may provide useful insights on how to integrate a major infectious disease, TB with a rising trend of NCDs.

## Conclusion

Gender, education and geographical location were significantly associated with the occurrence of at least one cardiometabolic risk factor among patients with TB in Nepal. The factors identified in this study can guide medical practitioners and policy makers to consider clinical suspicion, diagnosis and treatment. Revised TB treatment guideline can benefit by integrating the management of NCDs in TB treatment centers. The socio-cultural entanglements of these factors and the increasing co-morbidity of cardiovascular diseases urge the need for a broader approach of management of life-style related behavior.

## Data Availability

All data related to this study are included in the manuscript.

## Abbreviations

AHA: American Heart Association
ADA: American Diabetes Association
AOR: Adjusted Odds Ratio
BMI: Body Mass Index
CI: 95% Confidence Interval
DOTS: Directly Observed Treatment Short Course
JNC: Joint National Committee for Hypertension
LMICs: Low income and middle income countries
MDR: Multi-Drug Resistant
PTB: Pulmonary Tuberculosis
NCDs: Non-Communicable Diseases
PHCs: Primary Health Care Centres
SD: Standard Deviation
TB: Tuberculosis
WHO: World Health Organization
